# Follicular thyroid carcinoma in a child presenting as autonomously functioning thyroid nodule

**DOI:** 10.1186/1687-9856-2015-S1-P104

**Published:** 2015-04-28

**Authors:** Kiranjit Joshi, George Sim, Francis Lannigan, Felicity Frost, Priyanthi Kumarsinghe, Elizabeth Thomas, Glynis Price, Hieu Nguyen, Gareth Baynam, Adrien Charles, Catherine Choong

**Affiliations:** 1 Department of Endocrinology and Diabetes, Princess Margaret Hospital, Perth, Western Australia, Australia; 2 Department of Surgical Services, Princess Margaret Hospital, Perth, Western Australia, Australia; 3 Department of Pathology/Histopathology, Path-West QE 2 Medical Centre, Perth, Western Australia, Australia; 4 Department of diagnostic imaging and nuclear medicine, Princess Margaret Hospital, Perth, Western Australia, Australia; 5 Endocrine Surgical Services, Sir Charles Gairdner Hospital, Perth, Western Australia, Australia; 6 Genetic Services of Western Australia, King Edward Memorial Hospital, Perth, Western Australia, Australia; 7 School of Paediatrics and Child Health, University of Western Australia, Perth, Western Australia, Australia; 8 Department of Pathology/Histopathology, Path-West, Princess Margaret Hospital, Perth, Western Australia, Australia

## 

Thyroid nodules are uncommon in children (1.5%). However, the incidence of thyroid carcinoma in childhood thyroid nodules (26.4%) is 3-4 folds higher than in adults. Autonomously functioning thyroid nodules (AFTN) or hot nodules account for less than 3% of hyperthyroidism in children and carry a small risk of malignancy (2-5%). The majority of malignant hot nodules are papillary thyroid carcinoma (57.1%) with follicular carcinoma reportedly comprising 36.4% of cases. A search of MEDLINE identified 7 paediatric cases of AFTN harbouring carcinoma. Here we report a child, presenting with hyperthyroidism and subsequently diagnosed with follicular thyroid carcinoma.

An 11 year old girl presented with a palpable right sided thyroid nodule and symptoms of hyperthyroidism consisting of tachycardia and anxiety. A thyroid ultrasound showed a solitary, minimally heterogenous nodule on the right, measuring 2.5x1.9x1.6 centimetres. It was hyperechoic to the remainder of thyroid gland and had increased intralesional and perilesional vascularity. A radionuclide scan demonstrated the nodule as hyperfunctioning “hot” nodule with no radiotracer uptake in the rest of thyroid. An ultrasound guided FNAC was performed and features consistent with a follicular neoplasm were identified. She underwent right hemi-thyroidectomy and histopathology was consistent with angioinvasive follicular thyroid carcinoma. No evidence of carcinoma was found in the rest of the thyroid following completion thyroidectomy. Additionally, her head circumference was >98^th^ percentile, a lipoma was present over her sacrum and MRI suggested avascular hamartoma over her right ankle, together raising the possibility of Cowden syndrome.

FNAC is warranted in all cases of solid thyroid nodules in children including hot nodules to help define the pathology. If inconclusive, surgical excision of lesion for histopathology should be considered. Familial cancer syndromes, which can include a hamartoma/tumour phenotype such as Cowden syndrome and other PTEN hamartoma/tumour syndromes, DICER1 syndrome and MEN2 are also possible, depending on the type of thyroid malignancy.

Written informed consent was obtained from the patient's parent or guardian for publication of this abstract and any accompanying images. A copy of the written consent is available for review by the Editor of this journal.

